# Mucin impedes cytotoxic effect of 5-FU against growth of human pancreatic cancer cells: overcoming cellular barriers for therapeutic gain

**DOI:** 10.1038/sj.bjc.6603972

**Published:** 2007-10-02

**Authors:** A V Kalra, R B Campbell

**Affiliations:** 1 Department of Pharmaceutical Sciences, Northeastern University, 360 Huntington Avenue, Bouvé College of Health Sciences, Boston, MA 02115, USA

**Keywords:** pancreatic cancer, MUC1 *O*-glycosylation, 5-fluorouracil, drug delivery

## Abstract

Mucins are high molecular weight glycoproteins expressed on the apical surface of normal epithelial cells. In cancer disease mucins are overexpressed on the entire cellular surface. Overexpression of MUC1 mucin in pancreatic tumours has been correlated with poor patient survival. Current chemotherapeutic approaches such as 5-fluorouracil (5-FU) has produced limited clinical success. In this study we investigated the role of mucin in cytotoxic drug treatment to determine whether the extracellular domain of mucin impedes cytotoxic drug action of 5-FU. Human pancreatic cancer cells revealed high and relatively moderate MUC1 levels for Capan-1 and HPAF-II, respectively, compared to MUC1 negative control (U-87 MG glioblastoma) that showed relatively non-specific anti-MUC1 uptake. Benzyl-α-GalNAc (*O*-glycosylation inhibitor) was used to reduce mucin on cell surfaces, and neuraminidase was used to hydrolyse sialic acid at the distal end of carbohydrate chains. Benzyl-α-GalNAc had no effect on cell morphology or proliferation at the concentrations employed. The inhibition of *O*-glycosylation resulted in significant 5-FU antiproliferative activity against Capan-1 and HPAF-II, but not against U-87 MG. However, the exposure of cells to neuraminidase failed to improve the cytotoxic action of 5-FU. Our experimental findings suggest that the overexpression of mucin produced by human pancreatic tumours might limit the effectiveness of chemotherapy.

Numerous methods have been advocated for the treatment of human pancreatic (mucinous) carcinoma. The American Cancer Society has reported that no single treatment satisfies all treatment guidelines for malignant pancreatic disease, nor offers any significant impact on patient survival (American Cancer Society, 2007). A combination of surgical and non-surgical practices may thus be required to successfully eradicate the human disease. In order to improve treatment approaches we must consider the biology of pancreatic tumours at the molecular, cellular and physiological levels.

With the exception of gemcitabine there are no compelling data to support the use of any other drug agent (alone or in combination) over the use of fluoropyrimidines with regard to patient survival. Fluoropyrimidines work by inhibiting thymidylate synthase, a rate-limiting enzyme in *de novo* pyrimidine deoxynucleotide biosynthesis (van Triest and Peters, 1999; Fishman and Wadler, 2001). Although 5-fluorouracil (5-FU) has demonstrated antitumor activity, poor drug selectivity and modest tumour response to therapy have limited overall clinical success (Khanna *et al*, 2006; Kurt *et al*, 2006). Since metastatic disease remains incurable, comprehensive efforts have been undertaken to optimise fluoropyrimidine therapy. To accomplish this goal, factors contributing to suboptimal tumour response must be identified and investigated. For this reason, we investigate the role of mucin in cancer chemotherapy at the cellular level.

Mucins are high molecular weight glycoproteins having oligosaccharides attached to a protein backbone core by *O*-glycosidic linkages, and are approximately 50–80% carbohydrates in terms of total molecular mass (Gendler *et al*, 1991; Ruddon, 1995; Yonezawa *et al*, 2002). Mucin is produced in a wide range of host tissues including the gastrointestinal tract, lungs, kidneys, ovaries, breast and pancreas (Gendler *et al*, 1991; Ho *et al*, 1993; Corfield *et al*, 2001). Under normal physiological conditions mucin plays a protective role in epithelial tissues, functioning in the renewal and differentiation of the epithelium and in the regulation of cell adhesion and effector cell function (Hollingsworth and Swanson, 2004). High-level expression of mucin is associated with metastasis and poor clinical outcome in patients diagnosed with pancreatic cancer (Kigure, 2006).

Analysis of normal and tumour-derived mucins revealed that the carbohydrate chains of mucins are shorter and more sialyated in developing tumours compared to normal tissues (Beum *et al*, 1999; Peracaula *et al*, 2005). The synthesis of mucin on the surface of normal epithelial cells is under strict regulation, but in tumours there is an overabundance of mucin mainly due to elevated expression of MUC1. MUC1 is structurally unique possessing a transmembrane domain and a larger extracellular domain made up of tandem repeats of 20 amino acids, and a cytoplasmic tail. The glycoprotein is present in as many as 30–100 cellular copies (Lan *et al*, 1990), and unlike high levels of soluble mucin (ie, MUC2), MUC1 mucin is predominately associated with the cell membrane (Perey *et al*, 1992; van de Wiel-van Kemenade *et al*, 1993).

The increased density of mucin located at the membrane surface produces a structural mesh capable of limiting interactions of tumour cell epitopes in immune recognition (Jentoft, 1990; van de Wiel-van Kemenade *et al*, 1993). The main focus of the following study was to show that the formidable extracellular domain of mucin impedes the cytotoxic activity of 5-FU against human pancreatic cancer cells.

In this study we optimised the concentration of benzyl-2-acetamido-2-deoxy-α-D-galactopyranoside (benzyl-α-GalNAc), a reagent used to inhibit the synthesis of mucin *O*-glycosylation of cellular models of human pancreatic cancer. We established a relationship between the overabundance of MUC1 with general susceptibility of cells to the effects of 5-FU. Combinations of differential interference contract microscopy (DIC) and fluorescence microscopy as well as cytotoxicity and immunofluorescence studies were used to determine the importance of mucin in cytotoxic drug therapy.

## MATERIALS AND METHODS

### Materials

The chemotherapeutic agent 5-FU, sulforhodamine B (SRB), benzyl-α-GalNAc, neuraminidase from *vibro cholerae* was purchased from Sigma-Aldrich (St Louis, MO). TCA (trichloroacetic acid) and 1% acetic acid was purchased from Fisher Scientific Company (Fair Lawn, NJ). Fluorescein isothiocyanate (FITC)-conjugated mouse anti-human CD227 (MUC1) monoclonal antibody was purchased from BD Pharmingen (San Jose, CA). FITC-conjugated *Maackia amurensis* lectin (MAA) was purchased from EY Laboratories, Inc (San Mateo, CA). Cell culture media Eagle's minimum essential medium (EMEM), and Iscove's modified Dulbecco's medium and trypsin-ethylenediaminetetraacetic acid were purchased from ATCC (Manassas, VA).

### Cell culture

The human pancreatic cancer cell lines Capan-1 (HTB-79) and HPAF-II (CRL-1997) were maintained in Iscove's modified Dulbecco's Medium and EMEM, respectively. The human brain cancer cell line U-87 MG (glioblastoma, astrocytoma grade III) was maintained in EMEM supplemented with 10% fetal bovine serum. All cell lines were grown in a humidified CO_2_ atmosphere at 37°C.

### Immunofluorescence staining of cells

Sterile coverslips were placed in a six-well plate (Corning, NY). Cells were next seeded at 5 × 10^5^ per ml in the same six-well plate. Following an incubation period of 24 h at 37°C, 4 μl of FITC-conjugated mouse anti-human CD227 (MUC1) monoclonal antibody was added to each well. Cells were incubated for an additional 24 h with antibody and washed with 1 × phosphate-buffered saline (PBS) to remove unassociated antibody. The coverslip from each well was mounted onto a glass microslide (Corning, NY) with fluor mounting media (Trevigen Inc, MD) (Lemken *et al*, 2007). Finally, the association of CD227 (anti-MUC1) antibody with cells was determined using a combination of fluorescence and DIC microscopic applications at × 20 magnification (Olympus BX61WI, Melville, NY).

### Cell toxicity studies using benzyl-α-GalNAc

Cells were seeded at 1 × 10^4^ per ml in 48-well plates. Following a 24 h incubation period at 37°C, cells were exposed to various concentrations of benzyl-α-GalNAc solution prepared in cell culture medium. After 72 h of cell exposure to benzyl-α-GalNAc, the percent of cell viability was determined using SRB assay as discussed elsewhere (Skehan *et al*, 1990; Kalra and Campbell, 2006), and calculated as follows: 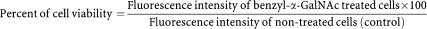 To observe the effects of benzyl-α-GalNAc exposure on cell morphology, DIC microscopy was employed. Sterile coverslips were placed in a six-well plate (Corning, NY). Cells were next seeded at 1 × 10^4^ per ml of culture medium in the six-well plate. Following a 24 h incubation period at 37°C the cells were exposed to the maximum non-toxic concentration of benzyl-α-GalNAc solution prepared in culture medium. Following 72 h of cell exposure to benzyl-α-GalNAc, each well was washed with 1 × PBS and the coverslip from each well was mounted onto a glass microslide with fluor mounting media. Finally, cell morphology was observed at × 20 magnification.

### RNA isolation and real-time RT–PCR

Cells were seeded at 2 × 10^4^ cells per ml of culture medium in a 24-well plate. Following a 24 h incubation period at 37°C, cells were exposed to maximum non-toxic concentration of benzyl-α-GalNAc prepared in required growth media. Forty-eight hours following exposure of cells to benzyl-α-GalNAc, the total RNA was extracted from the cells using RNA-STAT-60 (TEL-TEST, Friendswood, TX) according to the manufacturer's instructions. The total RNA extracted from cells was reverse transcribed to synthesise cDNA using SuperScript first-strand synthesis kit (Invitrogen Life Technologies, Carlsbad, CA). The total RNA was reverse transcribed in a final reaction volume of 20 μl, using random hexamers for 10 min at room temperature, 1 h at 42°C and 15 min at 70°C. Real-time PCR was performed using SYBR Green master mix (Applied Biosystems, Foster City, CA). The primer sequences used were: MUC1 (forward) GTGCCCCCTAGCAGTACCG, MUC1 (reverse) GACGTGCCCCTACAAGTTGG (de Cremoux *et al*, 2000), L32 mRNA was the internal standard with primer sequence, AGCAACAAGAAAACCAAGCACAT and TTGACATTGTGGACCAGGAACT (Lukashev *et al*, 2006). The real-time PCR was performed using Applied Biosystems 7300 Real-Time PCR system (Foster City, CA).

### Anti-MUC1 antibody (CD227) association studies

Cells were seeded at 2 × 10^4^ per ml of media in a 24-well plate. Following a 24 h incubation period at 37°C, cells were exposed to the maximum non-toxic concentration of benzyl-α-GalNAc (as shown in Figure 2A) for 24, 48 and 72 h. After each exposure time point the cells were washed with 1 × PBS and 1 ml of fresh media was added to each well. The cells were now exposed to 4 μl per well of CD227 antibody. Following 24 h of incubation, cells were washed with 1 × PBS to remove any unassociated antibody. Fluorescence intensity was measured using a fluorescence microplate reader (Bio-Tek Instruments Inc, VT) at excitation and emission wavelengths of 485 nm and 528 nm, respectively.

### Fluorescence-activated cell-sorting analysis

Cells were seeded at 2 × 10^4^ per ml in a 24-well plate. Following a 24 h incubation period at 37°C, cells were exposed to the maximum non-toxic concentration of benzyl-α-GalNAc as shown in Figure 2A. Forty-eight hours following exposure of cells to benzyl-α-GalNAc, the cells were next washed two times with 1 × PBS and 1 ml of fresh culture medium was added to each well. Next, cells were exposed to 4 μl per well of CD227 antibody. Following 24 h incubation with antibody, cells were washed with 1 × PBS to remove any unassociated antibody, and then detached from plate using 0.5 ml per well of trypsin. Finally, cells were washed with 1 × PBS and antibody association was determined by fluorescence-activated cell sorting (FACS) analysis (BD FACSCalibur, San Jose, CA, USA).

### Effect of inhibiting *O*-glycosylation with benzyl-α-GalNAc on cytotoxicity of 5-FU

Cells were seeded at 1 × 10^4^ per ml in a 48-well plate. Following a 24 h incubation period at 37°C, one row of cells was exposed to a predetermined concentration of benzyl-α-GalNAc solution prepared in growth medium, and another without the glycosylation inhibitor. Following additional 48 hours of incubation each row was washed with 1 × PBS and treated with 5-FU prepared in cell growth medium. The growth inhibitory effect of 5-FU after 24 h of exposure was determined by SRB assay and percent of viable cells was calculated as follows: 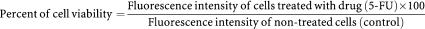


### Cell toxicity studies post-removal of benzyl-α-GalNAc

Cells were seeded at 1 × 10^4^ per ml in 48-well plates. Following a 24 h incubation period at 37°C, cells were exposed to the maximum non-toxic concentration of benzyl-α-GalNAc solution prepared in media. Following 48 h exposure to benzyl-α-GalNAc, cells were washed with 1 × PBS and then allowed to grow for an additional 24, 48 and 72 h in fresh culture medium. We determined percent of cell viability and investigated cell morphology 24 to 72 h post-removal of benzyl-α-GalNAc by SRB assay and DIC microscopy, respectively.

### Effect of neuraminidase action on cytotoxic effect of 5-FU

HPAF-II cells were seeded at 1 × 10^4^ per ml in 48-well plates. Following 24 h incubation period at 37°C, cells were exposed to neuraminidase (0.05 U ml^−1^) for 1 h. The removal of sialic acid residues from cell surface was confirmed by exposing cells to 0.05 U ml^−1^ neuraminidase for 1 h and labelled with 5 μl per well FITC-conjugated MAA lectin for 30 min followed by FACS analysis. To determine the effect of neuraminidase on CD227 association, cell exposed to neuraminidase were labelled with 4 μl per well of CD227 antibody for 4 h and antibody association determined using FACS analysis. Finally, cells exposed to 0.05 U ml^−1^ neuraminidase and were then treated with 5-FU (prepared in required growth medium) for 24 h. Percent cell viability was determined by SRB assay.

### Statistical analysis

To determine the significant difference between experimental groups, nonparametric Mann–Whitney *U*-test was used. Statistical significance was established at *P*⩽0.001. Analysis was performed using the statistical package SPSS 12.0 (SPSS Inc, Chicago, IL).

## RESULTS

### Immunofluorescence: relative sensitivity and selectivity of MUC1 (CD227) antibody for human pancreatic cancer cells

MUC1 is heterogeneously expressed in various physiological states. The structural mesh of mucin produced by cells in mucinous carcinomas (such as breast and pancreatic cancer) has been suggested to limit immune cell recognition by blocking the infiltration of lymphocytes in tumour tissues (van de Wiel-van Kemenade *et al*, 1993).

**Figure 1:**
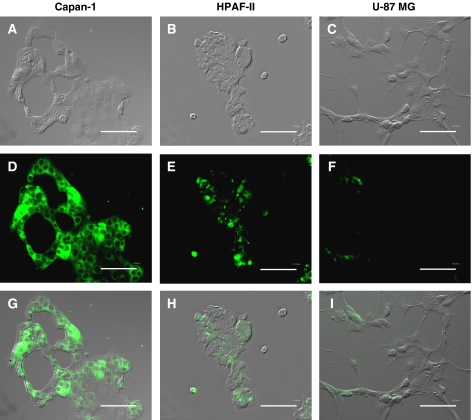
Immunofluorescence staining of cells using anti-MUC1 antibody (CD227). (**A**, **B)** DIC microscopy images show clusters of Capan-1 and HPAF-II cell lines and (**C**) DIC images show U-87 MG (negative control) cells without clustering. (**D**, **E**, **F**) Fluorescence microscopy images show relative extent of FITC-conjugated anti-MUC1 antibody (CD227) associated with cells. (**G**, **H**, **I**) Superimposed images confirm areas of antibody location with respect to each cellular cluster (× 20 magnification).

We investigated the role of membrane-associated mucin during cytotoxic drug treatment. We first confirmed whether anti-MUC1 antibody (CD227) is reliable enough to report changes in antibody binding to the peptide core whether in response to inhibition of *O*-glycosylation, or shedding of glycosylated functional groups from the core protein. We therefore evaluated the interaction of CD227 against two mucin-expressing human pancreatic cancer cell lines (Capan-1 and HPAF-II), and one (non-mucin expressing) human glioblastoma (U-87 MG) cell line as a negative control. Each cell line was incubated with FITC-conjugated CD227 and subsequently analysed by DIC and fluorescence microscopy to establish sensitivity and selectivity levels of CD227. DIC images show tightly joined clusters of Capan-1 and HPAF-II cells; images of regions surrounding individual cells were difficult to acquire (Figure 1A and B). Figure 1C shows a uniform arrangement of U-87 MG cells. Cells were more uniform in shape compared to Capan-1 and HPAF-II with limited intercellular associations; no cell clustering was observed.

Fluorescence images acquired for the interaction of CD227 with Capan-1 cells showed areas of intense antibody association, compared to relatively low uptake by HPAF-II and non-specific interaction with U-87 MG (Figure 1D–F). Capan-1 and HPAF-II cells have been shown elsewhere to produce heavy and moderate levels of MUC1, respectively. Our antibody-cell recognition studies involving the use of CD227 support other published reports (Sipos *et al*, 2003).

CD227 accurately distinguished between high and relatively low MUC1 mucin expression, and therefore represents a sensitive tool to evaluate the role of MUC1 during cytotoxic drug therapy. Relative to Capan-1 and HPAF-II, U-87 MG failed to recognise CD227, suggesting that CD227 can be used as a selective indicator of MUC1 positive expression for remaining studies (Figure 1G–I). Moreover, the distribution of MUC1 was generally observed on the entire surface of human pancreatic cells, and our CD227 reactivity studies involving Capan-1 show MUC1 mucin on Capan-1 cell membranes surrounding the cytoplasmic and nuclear cell compartment (Figure 1G).

### Inhibiting mucin *O*-glycosylation with benzyl--GalNAc: determination of maximum non-toxic concentration

**Figure 2:**
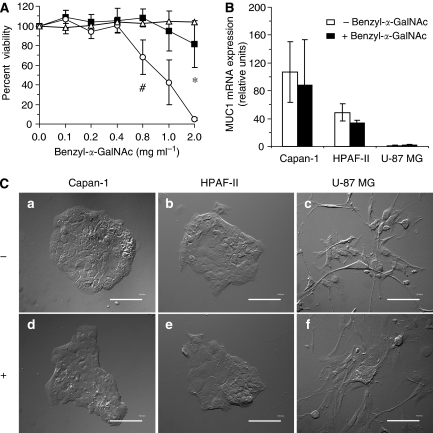
Determining the maximum non-toxic concentration of benzyl-α-GalNAc. (**A**) Percent cell viability of Capan-1 (○), HPAF-II (▪) and U-87 MG (Δ) cells was determined after exposing 1 × 10^4^ cells per ml to different concentrations of benzyl-α-GalNAc for 72 h. Percent viability of Capan-1 cells was significantly lower at concentrations greater than 0.4 mg ml^−1^ (^#^*P*<0.05 *vs* 0.8 mg ml^−1^); whereas the viability of HPAF-II cells was significantly lower beyond 0.8 mg ml^−1^ concentration (^*^P<0.05 *vs* 2.0 mg ml^−1^); no cell death was observed for U-87 MG cells upto 2.0 mg ml^−1^ concentration. (**B**) Real-time RT–PCR showed MUC1 mRNA expression levels for Capan-1 >HPAF-II >U-87 MG. The exposure of these cells to benzyl-α-GalNAc (+) did not induce alteration in MUC1 mRNA expression levels when compared to cells not exposed to benzyl-α-GalNAc (−). (**C**) DIC microscopy images show morphology of cells that were not exposed to benzyl-α-GalNAc (−) compared to structure of cells following 72 h exposure to benzyl-α-GalNAc (+) (0.4 mg ml^−1^ for Capan-1; 0.8 mg ml^−1^ for HPAF-II and U-87 MG) (× 20 magnification).

*O*-glycosylation is required for mucin formation. The elongation of *O*-glycosylated chains of the peptide core can be blocked by culturing mucin secreting cells with benzyl-2-acetamido-2-deoxy-α-D-galactopyranoside (benzyl-α-GalNAc) (Kuan *et al*, 1989). Incubation of SUIT-2 (human pancreatic cancer cell line) with 5 mM of benzyl-α- GalNAc for 72 h resulted in the inhibition of mucin *O*-glycosylation (Kuan *et al*, 1989). In order to determine the contribution of MUC1 expression in cytotoxic drug treatment, we first determined the maximum non-toxic concentration of benzyl-α-GalNAc that could be used to inhibit *O*-glycosylation of MUC1 without causing unwanted cellular death. Results from our cellular toxicity studies revealed a maximum concentration of 0.4 and 0.8 mg ml^−1^ of benzyl-α-GalNAc for the pancreatic cancer cells Capan-1 and HPAF-II, respectively (Figure 2A). The U-87 MG non-mucin-expressing cell line tolerated >0.8 mg ml^−1^. We performed real-time reverse transcription (RT)–PCR to determine whether treatment with benzyl-α-GalNAc would affect the transcript levels of MUC1 mRNA. Capan-1 cells showed high levels of MUC1 mRNA (106.8±43.5) as compared to HPAF-II cells (49±12.2), whereas the levels observed for negative control cell line U-87 MG were reported at 1.1±0.4 (Figure 2B). None of the cell lines exposed to benzyl-α-GalNAc exhibited any significant change in levels of MUC1 mRNA expression. We also observed morphological structures of cells after 72 h exposure to benzyl-α-GalNAc and observed no change in cell morphology compared to controls (Figure 2C). Based on these findings, the cell lines varied in their ability to tolerate effects of the inhibitor, and the corresponding non-toxic concentrations did not correlate with mucin expression levels.

### Confirming the inhibition of MUC1 *O*-glycosylation: determination of alterations in anti-MUC1 antibody association

**Figure 3:**
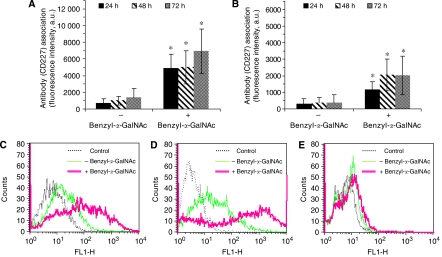
Inhibition of MUC1 mucin *O*-glycosylation. Approximately 2 × 10^4^ cells per ml of media were exposed to benzyl-α-GalNAc for 24, 48 and 72 h followed by 24 h incubation with FITC-conjugated anti-MUC1 (CD227) monoclonal antibody (4 μl well^−1^) at 37°C. The relative fluorescence intensities correlate with affinity of cells pretreated with benzyl-α-GalNAc (+) for CD227 compared to control (benzyl-α-GalNAc (−)) cells. Fluorescence intensities for antibody associated with (**A**) Capan-1 and (**B**) HPAF-II cells exposed to benzyl-α-GalNAc was significantly higher for 24, 48 and 72 h exposure time (^*^P⩽0.001) as compared to control (benzyl-α-GalNAc (−)). (**C–D)** Fluorescence-activated cell-sorting analysis for (**C**) Capan-1, (**D**) HPAF-II and (**E**) U-87 MG when exposed to benzyl-α-GalNAc (+) and compared to cells that were not previously exposed to benzyl-α-GalNAc. The specific peaks and lines to which the peaks are assigned are in the insert of the panel for FACS analysis.

The *O*-glycan chains present on MUC1 mucin can restrict access of the peptide core to antibodies (Ho *et al*, 1995). In order to determine whether the concentrations of benzyl-α-GalNAc employed are sufficient to inhibit MUC1 *O*-glycosylation, we observed association of CD227 antibody with cells immediately following exposure to the inhibitor. Our results showed that antibody association with both Capan-1 (Figure 3A) and HPAF-II (Figure 3B) cell lines was significantly enhanced (^*^P⩽0.001) after 24, 48 or 72 h exposure to benzyl-α-GalNAc (+) compared to control (benzyl-α-GalNAc (−)). Similarly, FACS analysis revealed a shift in fluorescence peaks for pancreatic cancer cells exposed to benzyl-α-GalNAc compared to the unexposed cell population (Figure 3 C and D), whereas no shift in peak was observed for the negative control cell line U-87 MG (Figure 3E). The non-toxic concentration of benzyl-α-GalNAc was capable of inhibiting MUC1 *O*-glycosylation, based on the extent to which the CD227 antibody interacted with MUC1 peptide recognition site of the protein core.

### Mucin is a cellular barrier limiting chemotherapeutic action of 5-FU against human pancreatic cancer cells

**Figure 4:**
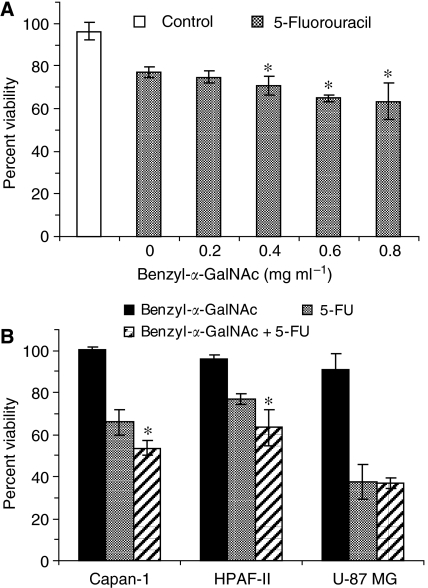
Effect of MUC1 mucin *O*-glycosylation on the cytotoxic action of 5-FU. We exposed (∼1 × 10^4^) cells to benzyl-α-GalNAc for 48 h followed by 24 h of treatment with 5-FU. (**A**) Percent viability of control cells (exposed to benzyl-α-GalNAc alone) was approximately 100%, whereas viability of HPAF-II cells exposed to benzyl-α-GalNAc followed by 5-FU treatment decreased with increasing concentrations of benzyl-α-GalNAc, and was significant at concentrations ⩾0.4 mg ml^−1^ (^*^P⩽0.001 *vs* 5-FU treatment alone). (**B**) Percent viability of Capan-1 and HPAF-II cells were significantly lower (^*^P⩽0.001) for cells exposed to benzyl-α-GalNAc followed by 5-FU treatment, compared to cells treated with 5-FU alone. The percent viability of U-87 MG cells (negative control) was similar for cells exposed to benzyl-α-GalNAc followed by 5-FU compared to 5-FU treatment alone.

Given the rapid turnover of mucin in adenocarcinomas, inhibition of *O*-glycosylation will probably occur only while cells are exposed to effect of inhibitor. Removal of benzyl-α-GalNAc will reactivate *O*-glycosylation synthesis, so the exposure of cells to 5-FU immediately following the removal of benzyl-α-GalNAc is key to maintaining the lowest possible levels of cellular-bound mucin. We first demonstrated the effects of increasing benzyl-α-GalNAc concentrations on cytotoxicity of 5-FU (108 μmol ml^−1^) against HPAF-II cells. The percent of cell viability was found to decrease with increasing concentrations of the inhibitor and this effect was significant (^*^P⩽0.001) at concentrations ⩾0.4 mg ml^−1^ as compared to 5-FU treatment alone (Figure 4A). The percent viability with 5-FU treatment alone was 77±2.5% which reduced significantly to 63±8.6% when pre-treated with the maximum non-toxic concentration of benzyl-α-GalNAc (0.8 mg ml^−1^). Similar results were observed for Capan-1 cells wherein the percent viability was 65±2% with 5-FU (80 μmol ml^−1^) alone, which was significantly (^*^P⩽0.001) lowered to 54±3.6% when exposed to benzyl-α-GalNAc (0.4 mg ml^−1^) followed by 5-FU treatment (Figure 4B). There was no significant difference in percent of cell viability of our control U-87 MG cells remaining at 37% when treated with 5-FU alone (100 μmol ml^−1^) or when exposed to benzyl-α-GalNAc (0.8 mg ml^−1^) prior to 5-FU treatment. These results suggest that the cytotoxic effect of 5-FU against MUC1-secreting cell lines was enhanced by inhibition of *O*-glycosylation.

### Effect of removing membrane-associated mucin: time-dependent analysis

**Figure 5:**
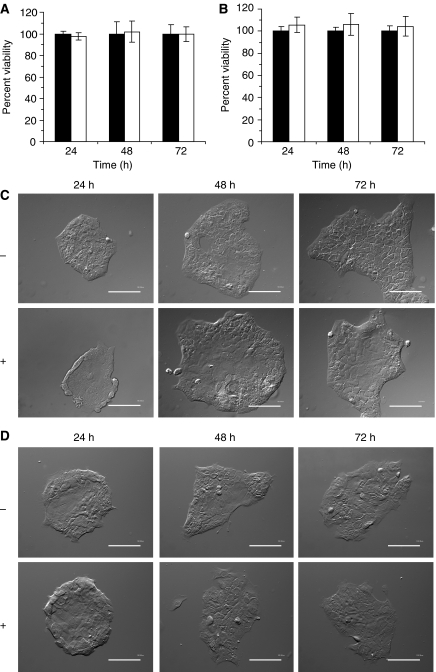
Cell proliferation and morphology post-removal of benzyl-α-GalNAc. (**A**) Capan-1 and (**B**) HPAF-II cells (1 × 10^4^ cells per ml) were exposed to 0.4 and 0.8 mg ml^−1^ of benzyl-α-GalNAc, respectively. Following 48 h exposure to benzyl-α-GalNAc the cells were washed with 1 × PBS and allowed to grow for next 24, 48 and 72 h in fresh media. The percent viability of cells was measured at each time point following exposure to benzyl-α-GalNAc (open bars, □) and compared with percent viability of cells not exposed to benzyl-α-GalNAc (closed bars, ▪). (**C**–**D**) Viability of Capan-1 and HPAF-II cells at 100% 24, 48 and 72 h post-removal of benzyl-α-GalNAc, and DIC microscopy of (**C**) Capan-1 and (**D**) HPAF-II cells post-removal of inhibitor (× 20 magnification).

Successful elimination of the glycosylated functional groups surrounding the MUC1 peptide core should not limit the capacity of cells to proliferate once benzyl-α-GalNAc has been removed. A significant change in cell's ability to multiply and divide due to exposure to benzyl-α-GalNAc would affect the measure of MUC1's role in our experiment. We next exposed Capan-1 and HPAF-II cells to benzyl-α-GalNAc for 48 h. Percent of cell viability was determined 24, 48 and 72 h after removal of benzyl-α-GalNAc. We observed no significant effect of inhibitor on growth of Capan-1 (Figure 5A) and HPAF-II (Figure 5B) cells post-removal of benzyl-α-GalNAc, compared to untreated controls. We next looked at DIC images of cells post-removal of benzyl-α-GalNAc and observed no visible morphological changes in cellular structures for both cell lines (Figure 5C and D).

On the basis that the rate of cellular proliferation is an accurate indicator of general cell health, our data supports the hypothesis that the concentration of benzyl-α-GalNAc selected for pre-treatment of Capan-1 and HPAF-II cells was non-toxic upto several days post-removal. Under these conditions the effect of chemotherapeutic agents (ie, 5-FU) against cells previously exposed to benzyl-α-GalNAc must be due to the chemotherapeutic agent alone, and not related to cellular exposure to benzyl-α-GalNAc.

### Effects of removing sialic acid residues on 5-FU cytotoxicity

In several pancreatic cancer cells the presence of terminal sialic acid sugar moiety on carbohydrate chains is higher due to the overexpression of sialyltransferases (Peracaula *et al*, 2005). We next determined whether the removal of the terminal sialic acid moiety of underglycosylated mucin sufficient to improve cytotoxic action of 5-FU, or is removal of the entire carbohydrate chain required.

**Figure 6:**
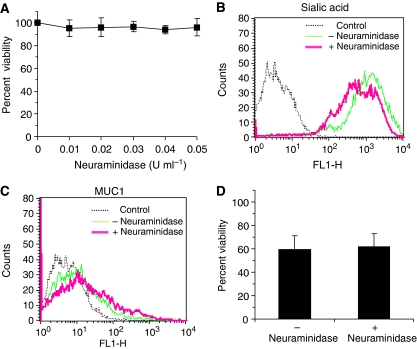
Effect of sialic acid removal on cytotoxic activity of 5-FU. Approximately 1 × 10^4^ HPAF-II cells per ml of growth medium were exposed to neuraminidase for 1 h at 37°C. (**A**) Cell viability was 100% upto 0.05 U ml^−1^ concentration of neuraminidase. (**B**) FACS analysis showing decrease in fluorescence intensity (correlating to association of FITC-conjugated MAA lectin to the sialic acid residues) for cells exposed to (+) neuraminidase compared to cells without exposure to (−) neuraminidase. (**C**) FACS analysis showing no change in fluorescence peaks for CD227 association with or without neuraminidase treatment. **(D**) When cells were previously exposed to neuraminidase (+), the effect of 5-FU on percent of cell viability was not significantly different from the control (neuraminidase (−)).

The enzyme neuraminidase was employed to cleave *N*-acetylneuraminic (or sialic acid), commonly associated with glycoproteins (Varki and Diaz, 1983). We observed that the concentrations of neuraminidase up to 0.05 U ml^−1^ had no effect on the growth of HPAF-II cells (Figure 6A). We next exposed these cells to (0.05 U ml^−1^ of) neuraminidase and confirmed the cleavage of sialic acid groups using FITC-conjugated MAA lectin, which binds to sialic acid residues (Wang and Cummings, 1988). FACS analysis revealed lower fluorescence intensities (shift in peak to the left) for cells exposed to neuraminidase compared to control without neuraminidase. Removal of sialic acid groups from underglycosylated carbohydrate chains was achieved (Figure 6B). We next observed that the exposure of cells to the action of neuraminidase did not significantly improve general access of CD227 to the MUC1 protein core (Figure 6C), and so the drug barrier effect of mucin was functionally intact in the absence of sialic acid. The next study explored the cytotoxic effect of 5-FU against cells previously exposed to neuraminidase. The effect of 5-FU against cells only or cells pretreated with neuraminidase was 59.3±11.8 and 61.7±11.1%, respectively (Figure 6D). We thus conclude that pre-treatment of cells with neuraminidase failed to influence general susceptibility of cells to the cytotoxic effects of 5-FU.

## DISCUSSION

The effectiveness of most chemotherapeutic agents depends on adequate intracellular uptake of drugs by tumour cells. Overexpression of certain cell membrane proteins such as p-glycoprotein and multidrug resistant proteins of cancer cells has been shown to contribute towards cellular resistance to chemotherapy by reducing intracellular drug uptake (Sharom, 1997). Several studies have identified mucins as one of the significant components of the cell surface glycocalyx in various tumour types such as colon, breast, lung and pancreas (Moniaux *et al*, 2004). We investigated the role of mucin in cytotoxic drug therapy using two cellular models of human pancreatic cancer. We selected our cell lines based on their ability to produce relative amounts of MUC1 mucin. For example, Capan-1 and HPAF-II have been reported to overexpress MUC1 mucin (Sipos *et al*, 2003); U-87 MG is negative for MUC1 expression as determined by FACS analysis using a multimodal imaging probe against MUC1 (Moore *et al*, 2004). The expression of MUC1 in normal epithelial cells is confined to the apical surface, but covers the entire surface of many cancer cells. Our immunofluorescence images support this observation: Capan-1 >HPAF-II >U-87 MG. The overexpression of MUC1 mucin has been shown to sterically hinder cell–cell and cell–matrix interactions (Wesseling *et al*, 1996). Moreover, the dense *O*-glycosylation on MUC1 can mask several cell surface receptors shown to reduce transfection efficiency (Stonebraker *et al*, 2004).

The extent to which the dense mucin mesh influences the antiproliferative activity of 5-FU was investigated using human pancreatic cancer cells. In several studies we used a FITC-conjugated CD227 antibody to probe the existence of a single mucin variety, MUC1. We note that other membrane-bound mucins such as MUC3, MUC4, MUC12, MUC16 and MUC17 are also present (Hollingsworth and Swanson, 2004; Moniaux *et al*, 2004); relative contribution to the functional mucin barrier effect is probably determined by their relative abundance and extended extracellular domain sequence.

It was reported that relatively high concentrations of 5-FU are required to achieve significant *in vitro* cytotoxic effects against Capan-1 and HPAF-II cells (Kalra and Campbell, 2006). Herein, we report the effect of inhibiting MUC1 *O*-glycosylation on the cytotoxic activity of 5-FU. We used benzyl-α-GalNAc (inhibitor of *O*-glycosylation) to reduce the mucin glycation mesh surrounding cells. The concentrations of benzyl-α-GalNAc employed for Capan-1 (0.4 mg ml^−1^) and HPAF-II (0.8 mg ml^−1^) had no effect on normal cell proliferation. Previous reports have shown that morphological changes in cells can result from exposure to benzyl-α-GalNAc. For example, Ls174T cells showed formation of intracellular cysts when exposed to benzyl-α-GalNAc for more than 3 days. Similar observations were made with HT-29 cells, which showed swelling of cells and accumulation of intracytoplasmic vesicles after prolonged exposure to the inhibitor (Leteurtre *et al*, 2003) however, Caco-2 cells showed no morphological changes. These studies suggested that the effect of benzyl-α-GalNAc is actually cell line dependent (Byrd *et al*, 1995; Leteurtre *et al*, 2003), and so concentration of inhibitor employed should vary according to cell type.

We exposed our cells to the maximum non-toxic concentration of benzyl-α-GalNAc for no more than 3 days. We observed no effect on cell morphology or in rates of proliferation during periods of cell exposure to benzyl-α-GalNAc, or days following the post-removal of reagent. The effect of benzyl-α-GalNAc on our cell lines was thus limited to the inhibition of mucin *O*-glycosylation alone, and did not include any unwanted cytotoxic effects at the concentrations employed. Previous reports have suggested that benzyl-α-GalNAc treatment can alter the apical expression of MUC1 on surface of HT-29 cells, whereas no such alteration was seen in Capan-1 cells (Leteurtre *et al*, 2003). The exposure of benzyl-α-GalNAc to cells used in this study did not induce alterations in MUC1 transcript expression, and therefore the exposure did not alter the overall expression of MUC1 on apical surface of these cells.

The ability of benzyl-α-GalNAc to inhibit MUC1 *O*-glycosylation has been confirmed previously using lectin labelling in HT-29 and Capan-1 cells (Leteurtre *et al*, 2003). The inhibition of MUC1 *O*-glycosylation in pancreatic cancer cells was confirmed by alterations in CD227 antibody association following exposure to benzyl-α-GalNAc. Both Capan-1 and HPAF-II cells showed higher CD227 antibody association after benzyl-α-GalNAc exposure. The concentrations of the inhibitor used was therefore sufficient to inhibit *O*-glycosylation, which in turn reduced the mucin mesh and improved the general access of CD227 to the (20 amino acid tandem repeats of the) MUC1 protein.

In order for 5-FU to exert its cytotoxic effect, a sufficient amount of drug must reach the intracellular compartment where 5-FU is converted to active metabolites (Weckbecker, 1991). The role of the mucus layer secreted on the normal intestinal epithelium in limiting diffusion of nutrients and small molecules has been discussed previously (Specian and Oliver, 1991). Studies outlined here support the role of mucin during cytotoxic drug therapy. The inhibition of mucin *O*-glycosylation enhanced the cytotoxic effects of 5-FU against human pancreatic cancer cell lines, but not against the mucin-deficient cell line. The exact mechanism is not known, but we speculate that the inhibition of mucin glycosylation may reduce the formidable mucin *O*-glycosylation mesh, facilitate the diffusion of drugs across the compromised mucus layer, improve intracellular drug uptake and enhance cytotoxic drug action. Interestingly, the removal of sialic acid residue present at the terminal end of *O*-linked carbohydrate chains did not improve the cytotoxic effect of 5-FU. The entire mucin carbohydrate chain may thus function in reducing intracellular uptake of 5-FU. Reducing or eliminating the functional barrier effect of mucin might provide opportunities to improve therapy.

On the basis of our findings, we report that the overexpression of MUC1 mucin on the surface of human pancreatic cancer cells impede the cytotoxic activity of 5-FU. Investigations into the role of mucin during chemotherapy in preclinical models are necessary to better understand the clinical implications of our experimental findings.
